# Special Issue “COVID-19: Diagnostic Imaging and Beyond”

**DOI:** 10.3390/jcm9092721

**Published:** 2020-08-24

**Authors:** Chiara Giraudo

**Affiliations:** Institute of Radiology, Department of Medicine—DIMED, University of Padova, 35122 Padova, Italy; chiara.giraudo@unipd.it; Tel.: +39-049-821-2357

Since the beginning of the severe acute respiratory syndrome coronavirus 2 (SARS-COV2) outbreak in China at the end of 2019, clinicians relied on diagnostic imaging to characterize the disease’s extension and severity [1]. In fact, chest x-ray is often not only the first-line imaging technique for patients with a clinical suspect or already a certain diagnosis at reverse transcriptase-polymerase chain reaction (RT-PCR) but it is also a reliable tool to monitor patients in critical condition [2]. The extensive use of chest x-rays and their efficiency is also demonstrated by the growing body of research proposing several scores based on this technique [3,4,5,6]. For instance, the score of Toussie et al. demonstrated good prognostic value, and the CARE (COVID-19 chest x-rAy scoRE) developed by our group, separately evaluating ground glass and consolidations, showed that the ground glass seems not to influence the outcome. Moreover, considering both features rather than ground glass or consolidations alone, better diagnostic performance was obtained for evaluations at admission only and with longitudinal data [6].

Certainly, CT allows a more detailed characterization of the disease. Indeed, signs like linear opacities, crazy paving, and the so called “spider web sign” can be easily detected [7,8]. Although most of the literature reports the use of non-contrast CT [9], since the vascular/endothelial injury caused by the virus has become evident [10], also pulmonary angiography CT has started to play a role in the diagnostic path of COVID-19 patients. For example, a single center study by Kaminetzky et al. including 62 COVID-19 positive patients reported 37.1% prevalence of pulmonary embolism [11]. Nevertheless, the current recommendation is to perform CT pulmonary angiography only in the case of worsening of the cardiorespiratory function or clinical suspicion of pulmonary embolism [9]. Further evidence in this direction is needed in order to establish a very accurate diagnostic algorithm for COVID-19 patients.

Considering the emerging evidence that the vascular injury acts at a multisystemic level, the role of other radiological techniques and the need of examining other anatomical areas in addition to the lungs should not be overlooked [12,13]. In fact, neurological symptoms are often reported and even a case of acute necrotizing hemorrhagic encephalopathy has been recently diagnosed [14] and cerebral microstructural changes described at MR imaging [15]. Moreover, intestinal ischemia and cholestasis have been identified on abdominal imaging of COVID-19 patients [16].

As it is well known, in the last decade, diagnostic imaging took advantage of the most recent technological developments, seeing rapid growth of machine learning and artificial intelligence applications. Such methods have started to be applied also for COVID-19. For instance, Ali et al. proposed a computer aided system to recognize COVID-19 infections and Homayounieh and colleagues demonstrated that radiomics features can help in predicting the outcome [17,18]. Considering this very promising evidence, future studies including clinical and laboratory variables in such models are highly recommended.

Despite the crucial role of diagnostic imaging in this pandemic, we should not underestimate the complex organizational mechanisms behind radiological examinations. In fact, in the last few months, radiological units had to be reorganized, aiming to guarantee the safety of in- and outpatients as well as of the entire staff [19,20,21]. The main challenge is surely represented by the execution of CT and MR scans, which usually require the transfer of patients, calling to action numerous healthcare professionals. As for previous epidemics like severe acute respiratory syndrome (SARS) and Middle East respiratory syndrome (MERS), some hospitals adopted portable CT scanners in emergency rooms (ER) or intensive care units (ICU) [22,23], whereas others used mobile devices on trucks [21]. In addition, because of such difficulties, ultrasound (US) was broadly used in the ER, COVID-19 wards, and ICU. Indeed, as demonstrated in several studies, clinicians and anesthesiologists successfully diagnosed and monitored COVID-19 patients, with the US reducing intra-hospital patient transfers [24,25,26].

The above-summarized evidence demonstrates that, since the declaration of the pandemic by the World Health Organization (WHO, Geneva, Switzerland) on 11 March 2020 [27], the experience shared by the scientific community worldwide gave us the chance to learn the typical features of COVID-19 in the acute and subacute phases. Nevertheless, much is still to be understood, especially about the long-term consequences of the disease, such as the occurrence of pulmonary fibrotic changes. Thus, I strongly believe that more needs to be done to face this challenge and support our clinicians in dealing with this complex disease.

This Special Issue is a chance for radiologists and clinicians to increase our knowledge about COVID-19, considering also its long-term effects. Review articles about the current evidence, as well as original articles presenting novel findings, will help us in improving our clinical practice. Research addressing also technical and organizational aspects that characterize the role of diagnostic imaging in the current pandemic will represent a source of growth for healthcare professionals.

As Guest Editor, I would like to sincerely thank all authors for their precious contributions and all readers for their interest in this Special Issue and for sharing the acquired knowledge.
